# Catastrophic health expenditure and its inequality in rural China: based on longitudinal data from 2013 to 2018

**DOI:** 10.1186/s12889-023-16692-7

**Published:** 2023-09-26

**Authors:** Weile Zhang, Min Su, Dongxu Li, Tianjiao Zhang, Wenhui Li

**Affiliations:** School of Public Administration, Inner Mongolia University, Zhaojun Road, Yuquan District, Hohhot, Inner Mongolia 010070 China

**Keywords:** Catastrophic health expenditure, Rural China, Inequality, Concentration index, Decomposition

## Abstract

**Background:**

Catastrophic health expenditure (CHE) is an important indicator of measuring health inequality. Previous studies mainly focused on specific vulnerable populations rather than a wider range of vulnerable areas through panel data. Rural China is often associated with an underdeveloped economy and insufficient health resources. This study aims to update the information on the extent of and trends in the incidence and inequality of CHE among the households of rural China through longitudinal survey data.

**Methods:**

Data were obtained from three waves of the China Health and Retirement Longitudinal Study (CHARLS): 2013, 2015, and 2018. In total, 2,575 households were included in the analysis. CHE was defined as household health expenditures exceeding 40% of non-food expenditures. Inequality in CHE was measured using the concentration curve and concentration index. The contribution to CHE inequality was decomposed using the concentration index decomposition method.

**Results:**

The incidence of CHE was 0.2341 (95% CI: 0.22, 0.25) in 2013, 0.2136 (95% CI: 0.20, 0.23) in 2015, and 0.2897 (95% CI: 0.27, 0.31) in 2018 in rural China. The concentration curve lay above the equality line, and the concentration index was negative: −0.1528 (95% CI: −0.1941, −0.1115) in 2013, −0.1010 (95% CI: −0.1442, −0. 0577) in 2015, and −0.0819 (95% CI: −0.1170, −0.0467) in 2018. Economic status, age, and chronic diseases were the main contributors to inequality in CHE.

**Conclusions:**

The incidence of CHE in rural China displayed an upward trend from 2013 to 2018, although it was not continuous. Furthermore, a strong pro-low-economic inequality in CHE existed in rural China. Mainly economic status, age, and chronic diseases contributed to this pro-low-economic inequality. Health policies to allocate resources and services are needed to satisfy the needs of rural households and provide more accessible and affordable health services. More concern needs to be directed toward households with chronic diseases and older persons to reduce the incidence of CHE and promote health equality.

**Supplementary Information:**

The online version contains supplementary material available at 10.1186/s12889-023-16692-7.

## Background

Catastrophic health expenditure (CHE) was defined as 40% or more of the household capacity to pay (CTP) expenditure allocated towards annual out-of-pocket (OOP) healthcare payments [1]. CHE may force households to sacrifice their basic healthcare necessities, sell assets, and even incur long-term debt [2–4]. Although China, as a major developing country, has made considerable progress in economic development and healthcare reform, CHE presents a concerning healthcare challenge for China [5–8].

Rural areas, in particular, are often associated with an underdeveloped economy and insufficient healthcare resources; therefore, CHE is also closely associated with rural households. An extensive literature shows that rural areas exhibited a considerable incidence of CHE [9–11], which negatively affected the quality of life and even trapped rural households in a vicious circle of “illness due to poverty and poverty caused by illness” [12, 13]. Previous studies and surveys have emphasized that income-related inequality in CHE in rural areas was more concentrated in the lowest-economic-status groups [14–16]. Moreover, a growing number of studies reported that economic status, education, lifestyle, and households with older persons with disabilities or chronic diseases were the main contributors to the occurrence of CHE inequalities in rural areas [17, 18].

These studies highlight the influence of CHE and prove that more efforts are required to mitigate CHE for rural households. Nevertheless, current studies have some shortcomings. First, most studies use cross-sectional data to analyze CHE in rural China and therefore cannot measure trends in the incidence of and inequality in CHE. Second, most studies rely on the Oaxaca–Blinder decomposition, concentration index and other single methods to analyze the inequality in CHE in rural areas and lack other methods to test for it. Third, most of the studies of CHE in rural areas lack relevant heterogeneity analysis of the concentration index and determinants in CHE to date. In light of these limitations, this study aimed to (1) update the information on the overall extent of and trends and income-related inequality in CHE in rural China using the China Health and Retirement Longitudinal Study (CHARLS) balanced panel data, (2) validate the robust of the results on income-related inequality using a concentration index method and the quantile regressions, (3) analyze the heterogeneity of inequality in rural China in order to analyze the differences between different populations in the CHE and its determinants, and (4) provide implementable recommendations for improving government policies and reducing the incidence of and inequality in CHE.

## Methods

### Ethics

The ethics review board of Peking University approved the CHARLS study (approval number IRB00001052–11015). Informed consent was obtained, and the data were anonymized for analysis.

### Data

**Fig.1:**
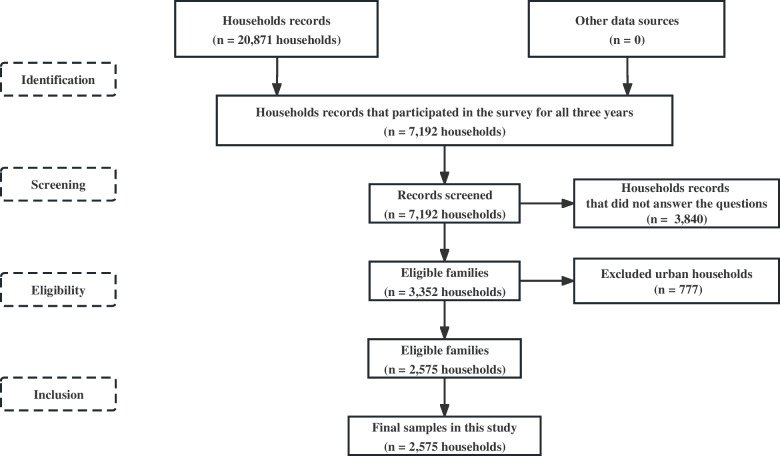
The process of screening participants in this study. Source: Author’s analysis

Data were derived from CHARLS, which covered 450 communities in 150 counties from 28 of the 32 provinces in mainland China. CHARLS, implemented by Peking University, aimed to collect a nationally representative sample of people aged 45 years or older to support aging and health-related research through a structured questionnaire [19] (the data and questionnaire are available at http://charls.pku.edu.cn/). After data cleaning, a total of 2,575 households were finally enrolled in this study. The detailed process is shown in Fig. 1.

### Measures

The CHARLS questionnaire includes basic personal information, household structure, health status, and other information. According to the questionnaire, rural households were defined by the item: “Was your address in the village or city/town?” The dependent variable was whether the household incurs CHE (a binary variable). The key independent variables include household head’s gender, age, marital status, education, insurance, smoking status, alcohol consumption, disability, chronic diseases, healthcare utilization, household size, and household economic status. It is important to note that the economic status of households was measured by dividing household income into five equal groups: lowest, lower, middle, higher, and highest.

The incidence of CHE refers to the ratio of households with CHE to all sample households in the survey [20]. The indicator determining whether CHE occurred was calculated as follows:

1 $$CHE=\left\{\begin{array}{c}1\, if\frac{OOP{ }_{h}}{CTP{ }_{h}}\gg 40\%\\ 0\, if\frac{OOP{ }_{h}}{CTP{ }_{h}}<40\%\end{array}\right\}$$

### Measuring CHE inequality

The concentration curve and concentration index were applied to analyze the inequality in CHE. The concentration curve and index were used to measure the extent of income-related inequality in the distribution of CHE across households [21]. When the concentration curve is above the line of equality, it denotes that inequality is concentrated in poor households; when it is under the line of equality, it denotes the opposite. The concentration index contributes to facilitating the identification of an effective way to reduce inequality. The concentration index ranges from −1 to 1, with a value of 0 indicating complete equality across income groups, and a positive concentration index denotes that households with high-economic-status are more likely to incur CHE than their low-economic-status counterparts, whereas a negative index denotes the opposite. The concentration index (CI) formula was as follows:

2 $$\left(\mathrm{C}=\frac{2}{\upmu }\mathrm{cov}\left({\mathrm{Y}}_{\mathrm{i}},{\mathrm{R}}_{\mathrm{i}}\right)\right)$$

where $cov$ is the covariance, ${Y}_{i}$ is the outcome variable, $\upmu$ is the mean of ${Y}_{i}$, and ${R}_{i}$ stands for the fractional ranks of household income. ${R}_{i}$ = i/N, i = 1 denotes the lowest-income households, and i = N denotes the highest-income households.

### Decomposition methods

Inequality can be further explained by decomposing the concentration index into its determining components. These determinants were selected according to previous research and constrained by the variables collected in the investigation [22]. Since CHE was a dummy variable, a probit model was employed to decompose and standardize the inequality in CHE. The regression model was as follows:

3 $$\mathrm{y}={\alpha }^{m}+{\sum }_{j}{\beta }_{j}^{m}{x}_{j}+{\sum }_{k}{\gamma }_{k}^{m}{Z}_{k}+\varepsilon$$

where ${\beta }_{j}^{m}$ and ${\gamma }_{k}^{m}$ are marginal effects, namely ${dy/dx}_{j}$ and ${dy/dz}_{k}$, and ε is the residual term. The decomposition result of the concentration index of the dependent variable $\mathrm{y}$ was as follows:

4 $$\mathrm{C}=\sum\nolimits_{j}\left({}^{{\beta }_{j}^{m}\overline{{x }_{j}}}\!\left/ \!{}_{\mu }\right.\right){C}_{j}+\left({}^{\overline{\varepsilon }}\!\left/ \!{}_{\mu }\right.\right){C}_{\mu }$$

where $\mathrm{C}$ is the concentration index of $\mathrm{y}$, $\upmu$ is the mean of $\mathrm{y}$, ${C}_{j}$ is the concentration index of ${x}_{j}$, ${C}_{\mu }$ is the concentration index of the residual term, and $\overline{{x}_{j}}$ and $\overline{\varepsilon }$ are the means of ${x}_{j}$ and $\varepsilon$, respectively.

### Analytical strategy

Categorical variables were presented as absolute numbers as proportions of the total number of participants. Logistic regression was employed to analyze the odds ratios (ORs) for CHE after controlling for several confounding factors at baseline, such as the year (2013, 2015, and 2018), gender, age, marital status, education, insurance, smoking status, alcohol consumption, disability, chronic diseases, health utilization, household size, and household economic status. All statistical analyses were performed using STATA statistical software version 15.1. A two-tailed *p*-value < 0.05 was considered statistically significant.

## Results

### Household descriptive statistics

The basic unit of analysis in this paper is the household. To reduce analytical error and accurately analyze the influencing factors of CHE, this study employed the information provided by the head of the household to represent the basic characteristics of the household, taking into account the practices of previous studies [23–25]. Table 1 presents a summary of the descriptive statistics for independent variables in 2013. A total of 48.85% of the heads of households were male, 65.75% were aged over 60 years, about 88% were living with a spouse, and the average household size was 1.69 people. In terms of economic status, 19.88%, 19.84%, 20.12%, 19.96%, and 20.19% of households had the lowest income, lower income, middle income, higher income, and highest income, respectively. Nearly 28% of the sample had junior high school-level education or above. An overwhelming majority of households were covered by insurance (96.23%), 61.59% of the household heads smoked, and nearly half consumed alcohol (48.66%). It was reported that 7% of the household heads had disabilities and 67% had chronic diseases. The sample’s annual outpatient and inpatient rates were 30% and 11%, respectively, and the annual outpatient time and inpatient time were 0.45 and 0.08 times, respectively.

**Table 1:** Basic characteristics of household heads in 2013 (*N* = 2,575)

**Variables**	**N (%)**	**Mean (S.D.)**
Sex		
Female	1317 (51.15)	
Male	1258 (48.85)	
Age		
≤ 50 years	47 (1.83)	
51–60 years	835 (32.43)	
61–70 years	1046 (40.62)	
≥ 71 years	647 (25.13)	
Marital status		
With spouse	2258 (87.69)	
Else (unmarried, divorced, widowed, etc.)	317 (12.31)	
Household size		1.69 (0.46)
Economic status		
Lowest	512 (19.88)	
Lower	511 (19.84)	
Middle	518 (20.12)	
Higher	514 (19.96)	
Highest	520 (20.19)	
Education		
Elementary school-level education and below	1865 (72.43)	
Junior high school-level education and above	710 (27.57)	
Insurance		
No	97 (3.77)	
Yes	2478 (96.23)	
Smoke		
No	989 (38.41)	
Yes	1586 (61.59)	
Drink		
No	1322 (51.34)	
Yes	1253 (48.66)	
Disability		
No	2397 (93.09)	
Yes	178 (6.91)	
Chronic diseases		
No	862 (33.48)	
Yes	1713 (66.52)	
Outpatient		
No	1813 (70.41)	
Yes	762 (29.59)	
Outpatient times		0.45 (1.32)
Inpatient		
No	2300 (89.32)	
Yes	275 (10.68)	
Inpatient times		0.08 (0.37)

Since there was no significant change in the descriptive data of the households from 2013 to 2018, only those from 2013 are presented; the standard deviation (S.D.) is shown in parentheses

### The incidence of CHE in rural China

**Fig. 2:**
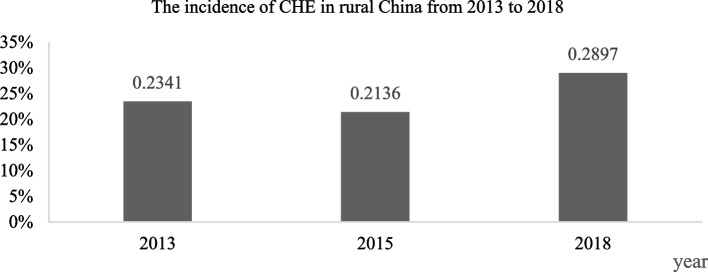
The incidence of CHE in rural China from 2013 to 2018. Source: Author’s analysis

Figure 2 illustrates the incidence of CHE from 2013 to 2018. The incidence of CHE was 0.2341 (95% CI: 0.22, 0.25) in 2013, 0.2136 (95% CI: 0.20, 0.23) in 2015, and 0.2897 (95% CI: 0.27, 0.31) in 2018.

### Determinants of CHE

A logistic regression analysis was conducted to analyze the factors influencing the occurrence of CHE in rural households. Table 2 presents the logistic regression analysis results of the longitudinal data. Compared with 2013, the incidence of CHE decreased approximately 0.87 times (95% CI:0.75, 1.00) in 2015 and increased 1.46 times (95% CI:1.27, 1.68) in 2018. The presence of older household members significantly increased the incidence of CHE. Specifically, households aged 61—70 years and older than 70 years were estimated to be 3.11 (95% CI: 1.56, 6.19) and 5.08 (95% CI: 2.54, 10.16) times more likely to incur CHE than households aged younger than 50 years, respectively. Compared with living with a spouse, not living with a spouse decreased the incidence of CHE approximately 0.69 times (95% CI: 0.52, 0.91). The economic status of households was another critical driver of CHE; for example, the richer and richest groups were 0.66 (95% CI: 0.52, 0.84) and 0.55 (95% CI: 0.44, 0.71) times less likely than the poorest group to suffer CHE, respectively. Those whose households had member(s) with chronic diseases were 1.52 (95% CI: 1.29, 1.79) times more likely to incur CHE than households with no chronic diseases. Regarding healthcare, those who used outpatient services and outpatient times were 1.41 (95% CI: 1.20, 1.66) and 1.10 (95% CI: 1.04, 1.15) times more likely than those who did not use such services to incur CHE, respectively.

**Table 2:** Determinants of the CHE using a logistic regression model (*N* = 2,575)

**Variables**	**Odds Ratio**	**95%C.I.**	
		lower-bound	upper-bound
Time (year)			
2013	1.00		
2015	0.87	0.75	1.00
2018	1.46	1.27	1.68
Sex			
Female	1.00		
Male	0.92	0.80	1.05
Age(years)			
≤ 50	1.00		
51–60	1.94	0.97	3.86
61–70	3.11	1.56	6.19
≥ 71	5.08	2.54	10.16
Marital status			
With a spouse	1.00		
Else (unmarried, divorced, widowed, etc.)	0.69	0.52	0.91
Household size	1.03	0.84	1.27
Economic status			
Lowest	1.00		
Lower	1.01	0.80	1.26
Middle	0.91	0.73	1.14
Higher	0.66	0.52	0.84
Highest	0.55	0.44	0.71
Education			
Elementary school-level education and below	1.00		
Junior high school-level education and above	0.87	0.73	1.05
Insurance			
No	1.00		
Yes	1.06	0.72	1.58
Smoke			
No	1.00		
Yes	0.93	0.79	1.09
Drink			
No	1.00		
Yes	0.79	0.68	0.92
Disability			
No	1.00		
Yes	1.16	0.88	1.54
Chronic diseases			
No	1.00		
Yes	1.52	1.29	1.79
Outpatient			
No	1.00		
Yes	1.41	1.20	1.66
Outpatient times	1.10	1.04	1.15
Inpatient			
No	1.00		
Yes	1.18	0.88	1.58
Inpatient times	1.20	0.94	1.52

*Abbreviations*: *CI* Contribution Index, *C.I.* Contribution Interval

### Concentration curve and index of CHE in rural China

**Fig. 3:**
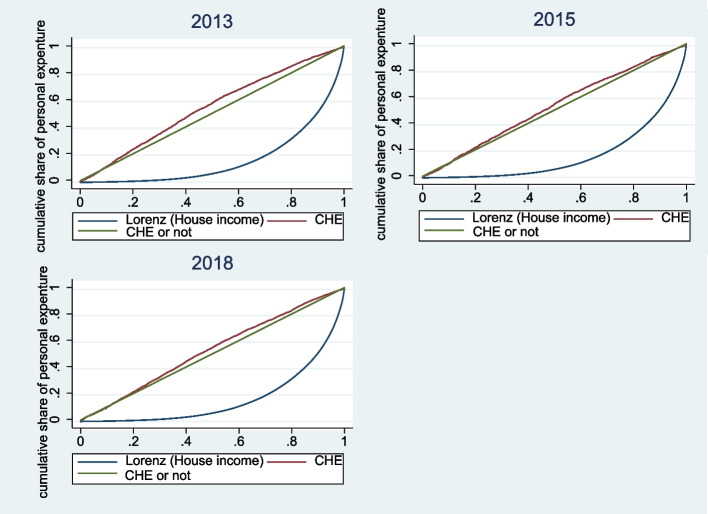
The concentration curves for rural households from 2013 to 2018. Source: Author’s analysis

**Fig. 4:**
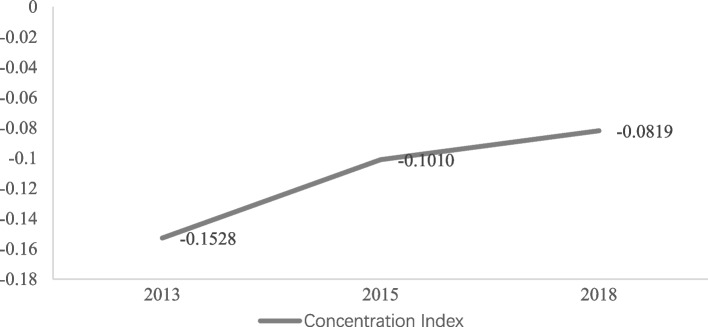
The concentration index of CHE in rural China from 2013 to 2018. Source: Author’s analysis

To analyze the inequality in the occurrence of CHE in rural households, the concentration curve and concentration index were drawn and calculated. Figure 3 shows that from 2013 to 2018, the concentration curve for rural households lay above the line of equality, indicating that CHE was more concentrated among low-economic- status households. Table 3 reveals the concentration index from 2013 to 2018. A positive concentration index indicates that rich households are more likely to incur CHE, whereas a negative index denotes the opposite. Overall, the concentration index for CHE was negative and decreased significantly from −0.1528 to −0.0764 for rural households from 2013 to 2018. The results were all negative, indicating that the inequality in CHE was mainly concentrated in poor rural households. Figure 4 presents the trend of the concentration index of CHE in rural China from 2013 to 2018. The concentration index was negative and showed a steady upward trend, indicating that inequality in the incidence of CHE is decreasing in rural areas.

**Table 3:** Concentration index of CHE in rural China from 2013 to 2018

**Time**	**Concentration Index (CI)**	**95% C.I.**	
		Lower-bound	Upper-bound
2013	−0.1528	−0.1941	−0.1115
2015	−0.1010	−0.1442	−0.0577
2018	−0.0819	−0.1170	−0.0467

*Abbreviations*: *CI* Contribution index, *C.I.* Contribution interval

### Results of quantile regression

To verify the robustness of the concentration index and avoid the effect of extreme values, the quantile regression model proposed by Koenker et al. was used for validation [26]. Table 4 shows the effect of household income on CHE at the 0.25, 0.50, and 0.75 quartiles. The results showed that CHE decreased significantly as household income increased, indicating that the higher the household income, the less likely CHE is to occur. Therefore, a pro-low-economic inequality in CHE existed in rural China.

**Table 4:** Quantile regression results of household income on CHE

**Variables**	**Household income**		
	**q25**	**q50**	**q75**
CHE	−494.786^***^ (−746.920, −242.652)	−2373.282^***^ (−3212.865, −1533.699)	−8668.390^***^ (−11,217.787, −6118.992)
Time	−11.263 (−75.056, 52.530)	−50.656 (−244.125, 142.812)	−0.000 (−509.719, 509.719)
Sex	1078.357^***^ (738.478, 1418.236)	2482.374^***^ (1445.865, 3518.884)	4349.501^***^ (1325.899, 7373.104)
Age	−822.740^***^ (−1030.431, −615.049)	−5640.978^***^ (−6469.727, −4812.228)	−9316.166^***^ (−11,366.295, −7266.037)
Marital status	−67.740 (−354.035, 218.554)	−1997.626^***^ (−3055.989, −939.263)	−9041.138^***^ (−13,370.558, −4711.717)
Household size	496.333^**^ (82.688, 909.978)	1257.709^**^ (241.380, 2274.039)	4608.026^**^ (291.260, 8924.792)
Education	742.783^**^ (142.485, 1343.082)	4759.358^***^ (2338.618, 7180.097)	10,116.805^***^ (6379.125, 13,854.486)
Insurance	462.538 (−96.275, 1021.350)	1493.687 (−366.315, 3353.690)	−445.164 (−7324.263, 6433.935)
Smoke	−210.922 (−572.380, 150.537)	−376.969 (−1554.915, 800.977)	−6121.501^***^ (−9351.603, −2891.400)
Drink	−469.957^***^ (−752.526, −187.387)	−2143.687^***^ (−3072.226, −1215.148)	−257.527 (−2848.546, 2333.491)
Disability	224.723 (−263.331, 712.776)	−2078.031^***^ (−3323.645, −832.417)	−12,213.725^***^ (−15,249.696, −9177.753)
Chronic diseases	−75.492 (−404.563, 253.579)	−1416.816^**^ (−2502.524, −331.108)	−2674.447^**^ (−5331.996, −16.898)
Outpatient	−34.781 (−344.076, 274.514)	151.061 (−804.651, 1106.774)	335.418 (−2779.481, 3450.317)
Outpatient times	−39.912 (−151.540, 71.717)	−84.427 (−385.065, 216.211)	−411.112 (−971.407, 149.184)
Inpatient	−340.898 (−921.293, 239.497)	−2732.556^***^ (−4713.183, −751.929)	−10,132.582^***^ (−13,930.479, −6334.685)
Inpatient times	573.311^**^ (5.934, 1140.688)	2560.279^***^ (819.132, 4301.427)	6755.444^***^ (3484.023, 10,026.865)
cons	25,875.340 (−102,818.275, 154,568.956)	123,530.642 (−265,988.612, 513,049.897)	52,240.473 (−976,195.222, 1,080,676.167)
N	7,725	7,725	7,725

95% confidence intervals in brackets, ^*^p < 0.1, ^**^p < 0.05, ^***^p < 0.01

### Decomposition of concentration index

To quantify the contribution of inequality to the occurrence of CHE in rural households, we conducted a concentration index decomposition analysis. Table 5 presents the contributions of each determinant to concentration index. A positive (negative) contribution denotes that the variable raised (reduced) the pro-high (low) economic inequality. In particular, we found that economic status, age, and having chronic diseases made the largest (73.50%, 63.26%, 57.72%), second largest (25.13%, 31.51%, 33.91%), and third largest (1.69%, 2.86%, 6.12%) contributions, respectively, to the inequality in CHE from 2013 to 2018.

**Table 5:** Decomposition of the concentration index of inequality in CHE, 2013–2018

**Variables**	**2013**		**2015**		**2018**	
	**Contribution to CI**	**Contribution to CI %**	**Contribution to CI**	**Contribution to CI %**	**Contribution to CI**	**Contribution to CI %**
Male	−0.0006	0.37	−0.0010	1.02	−0.0007	0.88
Age						
51–60 years	0.0179	−11.68	0.0322	−31.90	0.0132	−16.13
61–70 years	−0.0040	2.62	−0.0049	4.83	−0.0026	3.19
≥ 71 years	−0.0522	34.19	−0.0591	58.58	−0.0384	46.85
Junior high school − level education and above	0.0012	−0.77	−0.0020	2.03	−0.0052	6.34
Marital status	0.0044	−2.86	0.0055	−5.41	0.0040	−4.85
Insurance	0.0002	−0.14	−0.0002	0.24	0.0002	−0.30
Household size	−0.0012	0.79	−0.0022	2.18	0.0036	−4.37
Economic status						
Lower	−0.0021	1.36	0.0043	−4.29	−0.0035	4.26
Middle	0.0001	−0.05	−0.0001	−0.05	0.0001	−0.10
Higher	−0.0235	15.41	−0.0192	19.06	−0.0146	17.81
Highest	−0.0868	56.78	−0.0490	48.54	−0.0293	35.75
Smoke	−0.0010	0.63	0.0003	−0.33	−0.0001	0.13
Drink	0.0000	−0.03	0.0000	−0.32	0.0000	0.05
Disability	−0.0002	0.14	−0.0004	0.35	−0.0009	1.10
Chronic diseases	−0.0026	1.69	−0.0029	2.86	−0.0050	6.12
Outpatient	−0.0006	0.38	−0.0004	0.38	−0.0002	0.30
Outpatient times	−0.0015	0.98	−0.0033	3.24	0.0001	−0.09
Inpatient	−0.0005	0.35	−0.0005	0.46	−0.0003	0.40
Inpatient times	0.0001	−0.07	0.0003	−0.30	0.0002	−0.24

*Abbreviation*: *CI* Contribution Index

### Heterogeneity analysis of the concentration index of CHE

Table 6 presents a heterogeneity analysis of inequality in the occurrence of CHE based on age, chronic diseases, and education. We chose 65 years as the threshold to conduct the heterogeneity analysis of age [27]. The results showed that the concentration index for households aged ≥ 65 years was −0.1332, −0.1004, and −0.0514 in 2013, 2015, and 2018, respectively. The corresponding indices for household heads aged < 65 years were -0.1120, -0.0413, and -0.0561. From 2013 to 2018, the concentration indices for households with members with chronic conditions were −0.1530, −0.0900, and −0.0721. In contrast, the concentration indices for households without chronic diseases were −0.1373, −0.1130, and −0.0867. Finally, the concentration indices for households that received junior high school-level education and above were −0.1359, −0.1010, and −0.0589, while those with elementary school-level education and below had concentration indices of −0.1779, −0.1475, and −0.1145.

**Table 6:** Heterogeneity analysis of the concentration index of CHE in rural China, 2013–2018

**Time**	**Age difference**			
	**≥ 65**		**< 65**	
	**CI**	**95% C.I**	**CI**	**95% C.I**
2013	−0.1332	(− 0.1825, −0.0839)	−0.1120	(−0.1900, −0.0499)
2015	−0.1004	(− 0.1535, −0.0474)	−0.0413	(−0.1130, 0.0302)
2018	−0.0514	(− 0.0939, −0.0089)	−0.0561	(−0.1149, −0.0027)
**Time**	**Chronic diseases difference**			
	**Yes**		**No**	
	**CI**	**95% C.I**	**CI**	**95% C.I**
2013	−0.1530	(− 0.2001, −0.1059)	−0.1373	(−0.2208, −0.0539)
2015	−0.0900	(− 0.1395, −0.0405)	−0.1130	(−0.2004, −0.0256)
2018	−0.0721	(− 0.1111, −0.0332)	−0.0867	(−0.1632, −0.0102)
**Time**	**Education difference**			
	**Junior high school-level education and above**		**Elementary school-level education and below**	
	**CI**	**95% C.I**	**CI**	**95% C.I**
2013	−0.1359	(0.1820, −0.0898)	−0.1779	(−0.2686, −0.0873)
2015	−0.1010	(−0.1442, −0.0577)	−0.1475	(−0.2445, −0.0504)
2018	−0.0589	(−0.0975, −0.0202)	−0.1145	(−0.1953, −0.0337)

*Abbreviations*: *CI* Contribution Index, *C.I.* Contribution Interval

### Decomposition of concentration index based on heterogeneity analysis

Tables 7 and 8 show the results of the concentration index analysis for age differences. Table 7 shows that economic status (95.41%, 88.75%, and 107.64%), marital status (− 6.28%, −7.81%, and −3.37%), and gender (0.97%, 0.91%, and 5.36%) were the top three determinants of inequality when the age of the household head was ≥ 65 years in 2013, 2015, and 2018, respectively. Table 8 shows that economic status (85.42%, 57.16%, and 64.25%), chronic diseases (5.03%, 8.54%, and 12.91%), and number of outpatient visits (2.99%, 18.50%, and −0.88%) were the top three determinants of inequality when the age of the household head was < 65 years in 2013, 2015, and 2018, respectively.

**Table 7:** Decomposition of the concentration index of inequality in the CHE for age ≥ 65 years, 2013–2018

**Variables**	**2013**		**2015**		**2018**	
	**Contribution to CI**	**Contribution to CI %**	**Contribution to CI**	**Contribution to CI %**	**Contribution to CI**	**Contribution to CI %**
Male	−0.0013	0.97	−0.0009	0.91	−0.0028	5.36
Junior high school-level education and above	0.0005	−0.37	−0.0019	1.90	−0.0019	3.65
Marital status	0.0084	−6.28	0.0078	−7.81	0.0017	−3.37
Insurance	0.0007	−0.51	0.0002	−0.22	0.0019	−3.67
Household size	−0.0051	3.83	−0.0082	8.21	0.0066	−12.77
Economic status						
Lower	0.0129	−9.68	0.0108	−10.79	−0.0001	0.15
Middle	−0.0110	8.23	−0.0023	2.30	0.0001	−0.10
Higher	−0.0430	32.29	−0.0381	37.90	−0.0257	49.99
Highest	−0.0860	64.57	−0.0596	59.34	−0.0296	57.60
Smoke	−0.0001	0.09	−0.0000	0.02	0.0000	−0.12
Drink	0.0021	−1.56	0.0014	−1.37	0.0010	−1.97
Disability	0.0001	−0.08	−0.0001	0.13	0.0001	−0.19
Chronic diseases	0.0000	−0.02	0.0001	−0.07	0.0001	−0.19
Outpatient	−0.0001	0.10	−0.0001	0.07	−0.0001	0.14
Outpatient times	0.0005	−0.41	−0.0001	0.14	0.0005	−0.88
Inpatient	0.0000	−0.00	−0.0000	0.03	0.0000	−0.02
Inpatient times	0.0005	−0.35	0.0001	−0.93	0.0005	−1.04

*Abbreviation*: *CI* Contribution Index

**Table 8:** Decomposition of the concentration index of inequality in the CHE for age < 65, 2013–2018

**Variables**	**2013**		**2015**		**2018**	
	**Contribution to CI**	**Contribution to CI %**	**Contribution to CI**	**Contribution to CI %**	**Contribution to CI**	**Contribution to CI %**
Male	0.0004	−0.29	−0.0018	4.37	0.0020	−3.52
Junior high school-level education and above	−0.0011	0.92	0.0007	−1.67	−0.0042	7.40
Marital status	−0.0017	1.46	0.0013	−3.19	0.0021	−3.69
Insurance	−0.0000	0.00	0.0003	−0.76	0.0002	−0.40
Household size	−0.0005	−0.46	0.0007	−1.61	0.0005	−0.86
Economic status						
Lower	−0.0379	31.58	−0.0087	21.01	−0.0107	19.01
Middle	−0.0092	7.63	−0.0104	25.18	0.0095	−17.02
Higher	−0.0027	2.23	−0.0001	0.21	−0.0067	11.93
Highest	−0.0528	43.98	−0.0045	10.76	−0.0282	50.33
Smoke	−0.0006	0.49	0.0006	−1.52	−0.0002	0.44
Drink	−0.0004	0.30	−0.0003	0.73	−0.0005	0.84
Disability	−0.0007	0.61	−0.0004	1.04	−0.0026	4.61
Chronic diseases	−0.0060	5.03	−0.0035	8.54	−0.0072	12.91
Outpatient	0.0004	−0.32	0.0004	−0.92	−0.0010	1.75
Outpatient times	−0.0036	2.99	−0.0077	18.50	−0.0002	−0.88
Inpatient	−0.0016	1.35	−0.0009	2.18	−0.0010	1.75
Inpatient times	−0.0000	0.01	−0.0001	0.36	−0.0001	0.21

*Abbreviation*: *CI* Contribution Index

With regard to chronic diseases differences, S1 shows that among households with chronic diseases, economic status (77.38%, 66.63%, and 68.04%), age (18.93%, 26.12%, and 35.41%), and marital status (−2.43%, 4.40%, and −5.94%) were the top three determinants affecting CHE inequality from 2013 to 2018. S2 shows that among households without chronic diseases, economic status (58.97%, 63.18%, and 50.95%), age (4.96%, 4.06%, and 1.63%), and marital status (− 3.37%, 7.22%, and −5.14%) were also the top three determinates affecting CHE inequality from 2013 to 2018; see the Additional file 1 for details.

Regarding education differences, S3 shows that among households receiving junior high school-level education and above, economic status (69.16%, 66.39%, and 51.82%), age (18.64%, 11.27%, and 18.34%), and chronic diseases (2.35%, 0.59%, and 8.53%) were the top three determinants of CHE inequality from 2013 to 2018. S4 shows that among households receiving elementary school-level education and below, economic status (79.34%, 64.50%, and 73.87%), age (20.17%, 39.19%, and 40.34%), and chronic diseases (0.89%, 2.18%, and 3.57%) were the top three determinates of CHE inequality from 2013 to 2018; see Additional file 1 for details.

## Discussion

This study updates the knowledge on the trends in the equality of CHE for rural China in two ways. First, we used large, nationally representative longitudinal survey data from the CHARLS to evaluate the overall incidence of and trends and inequality in CHE from 2013 to 2018; thus, the findings are more generalizable to rural China and might help suggest a more apparent trend of CHE. Second, we conducted a heterogeneous decomposition analysis of the concentration index for CHE from 2013 to 2018 in rural China, facilitating the identification of an effective way to reduce inequality. Our study has three main findings.

First, we observed that the incidence of CHE in rural households increased from 0.2341 to 0.2897, although the trend was not continuous. The incidence of CHE in rural households increased from 2013 to 2018, which was consistent with the previous study [12, 16, 28]. One possible explanation is that OOP healthcare expenditure remains relatively high in China. As a consequence, patients and their households have greater financial risk and a higher probability of incurring CHE [29, 30]. Another possible explanation is the increased incidence of CHE due to the reimbursement policy of insurance system, which increases the need for medical care and direct and indirect medical expenditure [16].

Second, the study found that economic status, age, chronic diseases, and healthcare utilization were the main factors influencing the occurrence of CHE, which was consistent with previous studies [31–33]. The potential reasons for this can be explained as follows: First, the older the person, the worse the health status and the higher the healthcare expenditure, leading to a higher the likelihood of incurring CHE. Second, the higher the economic status, the more resistance to CHE; therefore, the lower the likelihood of CHE occurring. Third, the likelihood of experiencing CHE since chronic conditions are inherently prone to deterioration, and complications, and require long − term adherence to treatment, which leads to a continuous increase in healthcare expenditure. And finally, the more health care services are used, the higher the likelihood of encountering CHE. Finally, the more healthcare services are used, the higher the likelihood of incurring CHE.

Third, we found that there existed a strong pro − low − economic inequality in CHE in rural China. Moreover, economic status, age, and chronic diseases were the three main contributors to the inequality in CHE in rural China. There are several possible explanations: First, the health expenditure of China’s over-60 years population was 1.6 times that of non-elderly people, which would place a heavy financial burden on their households and society. In addition, as the human epidemiological spectrum has changed, chronic diseases have become one of the major threats to health. The Report on Nutrition and Chronic Diseases in China (2020) reported that the incidence of hypertension and diabetes in Chinese residents aged 18 years and above was 27.5% and 11.9%, respectively. Moreover, older persons have always been more susceptible to chronic diseases than their younger counterparts [34], therefore, there is an additive effect, especially in rural China.

Fourth, the heterogeneity analysis of CHE inequality by age, chronic disease, and education showed that CHE inequality was stronger for household heads aged ≥ 65 years than for those aged < 65 years. In addition, CHE inequality was also stronger for households without chronic diseases than for those with chronic diseases. Moreover, CHE inequality was stronger for households with elementary school-level education and below than for those with junior high school-level education and above. The finding may be explained by the following reasons. As in previous studies, health was inversely related to age and the likelihood of physical illness increases with age [35]. Therefore, as individuals age, the likelihood of medical expenditure increases, and the elderly are more likely to experience CHE in rural China. In China, the government provides targeted health management measures for patients with chronic diseases, such as the provision of chronic diseases management services [36, 37], which can effectively reduce healthcare costs and the incidence of CHE in rural China. In addition, the higher the level of education, the greater the focus on health status. Higher educated people tend to prioritize disease prevention, which effectively reduces the cost of possible subsequent treatment, and are therefore less likely to experience CHE [38].

Finally, the results showed that the medical insurance system had little statistical significance for CHE and did not reduce the financial burden on rural households. This finding was similar to previous reports [39, 40]. The possible explanations are as follows: First, only 3.77% of the 2,575 households in the study sample were not enrolled in any health insurance scheme; therefore, the smaller sample made the variable insignificant for CHE. Second, although the medical insurance system reduces the burden of health care costs, it stimulates the demand for health care and, therefore, increases health care expenditure.

### Strengths and limitations

This study has several strengths. First, this study measured the current status of CHE and inequality trends in rural China using balanced panel data from the CHARLS, which can provide a more comprehensive representation of CHE in rural China. Second, this study used a concentration index method and quantile regression to calculate and validate the results on inequality in CHE. Finally, this study employed a heterogeneity analysis of the inequality in the occurrence of CHE in rural China, which provides a theoretical basis for targeted improvements in Chinese health policy.

This study also has some limitations. First, the data were self-reported and limited by the pre-specified questions, personal preferences, and recall bias, which might make them prone to measurement errors. Additionally, the availability of measurement determinants for CHE was limited by the pre-specified questions in the survey, such as the failure to account for rural households that did not seek or gave up treatment due to the inability to pay, and the indirect opportunity cost caused by care, which may lead to an underestimation of the incidence of and inequality in CHE. Furthermore, although this analysis covered CHE in 2013, 2015, and 2018 in rural China, it was not continuous; hence, the data might not be comprehensive enough to identify the changes in the inequality in CHE. As continuous waves are to be added in the future, it will be important to reexamine these trends.

## Conclusions

The results showed that the incidence of CHE in rural China displayed an upward trend, although it was not continuous, and its inequality in CHE was mainly focused on the pro-low-economic households. In addition to economic status, age and chronic diseases were the main contributors to this pro-low-economic inequality. Moreover, heterogeneity differences in CHE inequality existed for age, chronic diseases, and education. Therefore, health policies to allocate accessible and affordable resources and services are needed to satisfy the needs of rural households, especially for lower-economic-status households. Additional strategies are needed to further reduce the socioeconomic differences and narrow the health gap between different income groups, and more attention needs to be directed toward households with chronic diseases and older persons.

### Supplementary Information

**Additional file 1: S1.** Decomposition of the concentration index of inequality of the CHE for people with chronic diseases, 2013–2018. **S2.** Decomposition of the concentration index of inequality of the CHE for people without chronic diseases, 2013–2018. **S3.** Decomposition of the concentration index of inequality in CHE for people with junior high school and above, 2013–2018. **S4.** Decomposition of the concentration index of inequality in CHE for people with elementary school and below, 2013–2018.

## Data Availability

This data was drawn from the Data were derived from the China Health and Retirement Longitudinal Study (CHARLS). They are opened to everyone. Researchers who want to use these data can visit http://charls.pku.edu.cn/. We had added questionnaires content in the Additional file 1.

## Abbreviations

CHE: Catastrophic health expenditure
CHARLS: China Health and Retirement Longitudinal Study
CTP: Capacity to pay
OOP: Out-of-pocket healthcare payments
CI: Concentration index
C.I.: Contribution interval
OR: Odds ratio

## References

[1] Xu K, Evans DB, Kawabata K, Zeramdini R, Klavus J, Murray CJ (2003). Household catastrophic health expenditure: a multicountry analysis. Lancet.

[2] Alam K, Mahal A (2014). Economic impacts of health shocks on households in low and middle income countries: a review of the literature. Glob Health.

[3] Loganathan K, Deshmukh PR, Raut AV (2017). Socio-demographic determinants of out-of-pocket health expenditure in a rural area of Wardha district of Maharashtra, India. Indian J Med Res.

[4] Li J, Jiao C, Nicholas S, Wang J, Chen G, Chang J (2020). Impact of medical debt on the financial welfare of middle- and low-income families across China. Int J Environ Res Public Health.

[5] Wagstaff A, Lindelow M (2008). Can insurance increase financial risk? The curious case of health insurance in China. J Health Econ.

[6] Xu X, Yang H (2022). Does elderly chronic disease hinder the sustainability of borderline poor families’ wellbeing: an investigation from catastrophic health expenditure in China. Int J Public Health.

[7] Sun Q, Liu X, Meng Q, Tang S, Yu B, Tolhurst R (2009). Evaluating the financial protection of patients with chronic disease by health insurance in rural China. Int J Equity Health.

[8] Gwatidzo SD, Stewart WJ (2017). Diabetes mellitus medication use and catastrophic healthcare expenditure among adults aged 50+ years in China and India: results from the WHO study on global AGEing and adult health (SAGE). BMC Geriatr.

[9] Abdel-Rahman S, Abonazel MR (2021). New measure of catastrophic health expenditures with application on rural Egypt. Middle East Dev J.

[10] Li Y, Wu Q, Gao L (2012). Analysis on causes of catastrophic health expenditure in rural China from perspective of system analysis. Chin J Health Policy.

[11] Li A, Shi Y, Yang X, Wang Z (2019). Effect of critical illness insurance on household catastrophic health expenditure: the latest evidence from the National Health Service Survey in China. Int J Environ Res Public Health.

[12] Sepehri A, Vu PH (2019). Severe injuries and household catastrophic health expenditure in Vietnam: findings from the Household Living Standard Survey 2014. Public Health.

[13] Okedo-Alex IN, Akamike IC, Ezeanosike OB, Uneke CJ (2019). A review of the incidence and determinants of catastrophic health expenditure in Nigeria: implications for universal health coverage. Int J Health Plann Manag.

[14] Borde MT, Loha E, Johansson KA, Lindtjrn B (2020). Financial risk of seeking maternal and neonatal healthcare in southern Ethiopia: a cohort study of rural households. Int J Equity Health.

[15] Chen RY, Yin AT, Zhao WJ, Han ZY, Wang WH, Li-Gang XU, Jing SS, Dong-Ping MA, Hou-Li XU, Hou J (2012). Research on the association of rural residents disease economic risk and disastrous health spending of Tengzhou City. Health Econ Res.

[16] Gu H, Kou Y, Yan Z, Ding Y, Shieh J, Sun J, Cui N, Wang Q, You H (2017). Income related inequality and influencing factors: a study for the incidence of catastrophic health expenditure in rural China. BMC Public Health.

[17] Brinda EM, Rajkumar AP, Enemark U, Prince M, Jacob KS (2012). Nature and determinants of out-of-pocket health expenditure among older people in a rural Indian community. Int Psychogeriatr.

[18] Xu X, Yang H (2022). Elderly chronic diseases and catastrophic health expenditure: an important cause of Borderline Poor Families’return to poverty in rural China. Humanit Soc Sci Commun.

[19] Zhao Y, Hu Y, Smith JP, Strauss J, Yang G (2014). Cohort profile: the China health and retirement longitudinal study (CHARLS). Int J Epidemiol.

[20] Xu K (2005). Distribution of health payments and catastrophic expenditures methodology.

[21] Mcgrail KM, Doorslaer EV, Ross NA, Sanmartin C (2009). Income-related health inequalities in Canada and the United States: a decomposition analysis. Am J Public Health.

[22] Liu S, Coyte PC, Fu M, Zhang Q (2021). Measurement and determinants of catastrophic health expenditure among elderly households in China using longitudinal data from the CHARLS. Int J Equity Health.

[23] Shikuro D, Yitayal M, Kebede A, Debie A (2020). Catastrophic out-of-pocket health expenditure among rural households in the semi-pastoral community, Western Ethiopia: a community-based cross-sectional study. Clinicoecon Outcomes Res.

[24] Servan-Mori E, Orozco-Nunez E, Guerrero-Lopez CM, Miranda JJ, Jan SP, Downey L, Feeny E, Heredia-Pi I, Flamand L, Nigenda G, Norton R, Lozano R (2023). A gender-based and quasi-experimental study of the catastrophic and impoverishing health-care expenditures in Mexican households with elderly members, 2000–2020. Health Syst Reform.

[25] Arenliu Qosaj F, Froeschl G, Berisha M, Bellaqa B, Holle R (2018). Catastrophic expenditures and impoverishment due to out-of-pocket health payments in Kosovo. Cost Eff Resour Alloc.

[26] Koenker R, Bassett GW (1978). Regression quantiles. Econometrica.

[27] Wang S. Spatial patterns and social-economic influential factors of population aging: A global assessment from 1990 to 2010. Social science & medicine. 2020;253:112963. 10.1016/j.socscimed.2020.112963. 10.1016/j.socscimed.2020.112963 32289647

[28] Xie B, Huo M, Wang Z, Chen Y, Fu R, Liu M, Meng Q (2018). Impact of the New Cooperative Medical Scheme on the trend of catastrophic health expenditure in Chinese rural households: results from nationally representative surveys from 2003 to 2013. BMJ Open.

[29] Wu Q, Li Y, Xu L (2012). Effect analysis on universal insurance coverage to reduce the incidence of catastrophic health expenditure in China. Chin J Health Policy.

[30] Mortensen K, Dean BE, French TM, Wang N, Chen J (2021). Trends in health care utilization and expenditures in the United States across 5 decades: 1977–2017. Med Care.

[31] Kien VD, Minh HV, Ngoc NB, Phuong TB, Quam MB (2017). Inequalities in household catastrophic health expenditure and impoverishment associated with noncommunicable diseases in Chi Linh, Hai Duong, Vietnam. Asia Pac J Public Health.

[32] Antunes FA, Jacobs B, De Groot R, Thin K, Hanvoravongchai P (2018). Equality in financial access to healthcare in Cambodia from 2004 to 2014. Health Policy Plan.

[33] Aregbeshola BS, Khan SM (2018). Determinants of catastrophic health expenditure in Nigeria. Eur J Health Econ.

[34] Fang EF, Xie CL, Schenkel JA, Wu C, Woo J (2020). A research agenda for ageing in China in the 21st century (2nd edition): focusing on basic and translational research, long-term care, policy and social networks. Ageing Res Rev.

[35] Klemenc-Ketis Z, Smogavec M, Softic N, Kersnik J (2011). Health-related quality of life: a population based study from Slovenia. Cent Eur J Public Health.

[36] Wang L, Liu W (2022). Effects of family doctor contract services on the health-related quality of life among individuals with diabetes in China: evidence from the CHARLS. Front Public Health.

[37] Wang Z, Li X, Chen M (2015). Catastrophic health expenditures and its inequality in elderly households with chronic disease patients in China. Int J Equity Health.

[38] Liu Q, Liu J, Sui S (2020). Public medical insurance and healthcare utilization and expenditures of older with chronic diseases in rural China: evidence from NRCMS. Int J Environ Res Public Health.

[39] Wei Y (2013). China’s new cooperative medical scheme and equity in access to health care: evidence from a longitudinal household survey. Int J Equity Health.

[40] Jing S, Yin A, Shi L, Liu J (2013). Whether new cooperative medical schemes reduce the economic burden of chronic disease in rural China. PLoS One.

