# Supplementary material for: Catastrophic health expenditure and its inequality in rural China: based on longitudinal data from 2013 to 2018
Source: BMC Public Health. 2023 Sep 26;23:1861. doi: 10.1186/s12889-023-16692-7 (PMC10521565; doi:10.1186/s12889-023-16692-7)
Supplement: Supplementary file 1 — Additional file 1: S1. Decomposition of the concentration index of inequality of the CHE for people with chronic diseases, 2013–2018. S2. Decomposition of the concentration index of inequality of the CHE for people without chronic diseases, 2013–2018. S3. Decomposition of the concentration index of inequality in CHE for people with junior high school and above, 2013–2018. S4. Decomposition of the concentration index of inequality in CHE for people with elementary school and below, 2013–2018. [file 12889_2023_16692_MOESM1_ESM.docx]

| **Variables** | **2013** | | **2015** | | **2018** | |
| --- | --- | --- | --- | --- | --- | --- |
|  | **Contribution to CI** | **Contribution to CI %** | **Contribution to CI** | **Contribution to CI %** | **Contribution to CI** | **Contribution to CI %** |
| Male | −0.0019 | 1.24 | −0.0012 | 1.38 | −0.0004 | 0.60 |
| Age |  |  |  |  |  |  |
| 51—60 years | 0.0083 | −5.41 | 0.0164 | −18.27 | 0.0010 | −1.44 |
| 61—70 years | 0.0027 | −1.75 | 0.0032 | −3.51 | 0.0016 | −2.23 |
| ≥ 71 years | −0.0399 | 26.09 | −0.0431 | 47.90 | −0.0282 | 39.08 |
| Junior high school−level education and above | 0.0001 | −0.98 | −0.0033 | 3.67 | −0.0031 | 4.23 |
| Marital status | 0.0037 | −2.43 | 0.0040 | −4.40 | 0.0043 | −5.94 |
| Insurance | 0.0002 | −0.19 | −0.00013 | 0.12 | −0.0001 | 0.19 |
| Household size | 0.0001 | −0.09 | −0.0015 | 0.12 | 0.0026 | −3.62 |
| Economic status |  |  |  |  |  |  |
| Lower | 0.0049 | −0.32 | −0.0001 | 0.08 | 0.0033 | −4.55 |
| Middle | −0.0095 | 0.62 | 0.0004 | −0.47 | −0.0012 | 1.72 |
| Higher | −0.0333 | 21.78 | −0.0195 | 21.72 | −0.0162 | 22.53 |
| Highest | −0.0846 | 55.30 | −0.0408 | 45.30 | −0.0349 | 48.34 |
| Smoke | −0.0010 | 0.64 | 0.0003 | −0.30 | −0.0003 | 0.38 |
| Drink | −0.0000 | 0.02 | −0.0000 | 0.02 | −0.0000 | 0.03 |
| Disability | −0.0008 | 0.61 | −0.0000 | 0.03 | −0.0009 | 1.25 |
| Outpatient | −0.0004 | 0.26 | −0.0003 | 0.33 | −0.0002 | 0.28 |
| Outpatient times | −0.0016 | 1.07 | −0.0030 | 3.35 | −0.0000 | 0.03 |
| Inpatient | 0.0000 | −0.00 | 0.0000 | −0.00 | 0.0000 | −0.00 |
| Inpatient times | 0.0006 | −0.38 | 0.0007 | −0.84 | 0.0005 | −0.68 |

S1 Decomposition of the concentration index of inequality of the CHE for people with chronic diseases, 2013–2018

Abbreviations: Contribution Index

S2 Decomposition of the concentration index of inequality of the CHE for people without chronic diseases, 2013–2018

| **Variables** | **2013** | | **2015** | | **2018** | |
| --- | --- | --- | --- | --- | --- | --- |
|  | **Contribution to CI** | **Contribution to CI %** | **Contribution to CI** | **Contribution to CI %** | **Contribution to CI** | **Contribution to CI %** |
| Male | −0.0004 | 0.32 | 0.0015 | −1.32 | −0.0014 | 1.64 |
| Age |  |  |  |  |  |  |
| 51—60 years | 0.3452 | −251.35 | 0.3811 | −337.11 | 0.3095 | −357.09 |
| 61—70 years | −0.1295 | 94.30 | −0.1422 | 125.78 | −0.1149 | 132.59 |
| ≥ 71 years | −0.2225 | 162.01 | −0.2435 | 215.39 | −0.1960 | 226.13 |
| Junior high school−level education and above | −0.0018 | 1.32 | 0.0053 | −4.73 | −0.0118 | 13.65 |
| Marital status | 0.0046 | −3.37 | 0.0082 | −7.22 | 0.0045 | −5.14 |
| Insurance | −0.0002 | 0.18 | −0.0009 | 0.81 | 0.0019 | −2.23 |
| Household size | −0.0053 | 3.89 | −0.0042 | 3.72 | 0.0046 | −5.33 |
| Economic status |  |  |  |  |  |  |
| Lower | 0.0079 | 5.72 | 0.0179 | −15.87 | −0.0220 | 25.39 |
| Middle | 0.0004 | −0.31 | −0.0011 | 0.96 | −0.0018 | 2.13 |
| Higher | −0.0007 | 0.51 | −0.0211 | 18.65 | −0.0087 | 10.01 |
| Highest | −0.0729 | 53.05 | −0.0672 | 59.44 | −0.0116 | 13.42 |
| Smoke | −0.0006 | 0.42 | 0.0004 | −0.34 | 0.0003 | −0.38 |
| Drink | 0.0008 | −0.57 | 0.0006 | −0.49 | 0.0006 | −0.80 |
| Disability | 0.0000 | −0.00 | −0.0000 | 0.01 | −0.0000 | 0.01 |
| Outpatient | 0.0014 | 0.10 | 0.0004 | −0.35 | 0.0001 | −0.15 |
| Outpatient times | −0.0000 | 0.03 | −0.0005 | 0.48 | 0.0008 | −5.33 |
| Inpatient | −0.0068 | 4.96 | −0.0020 | 1.77 | −0.0031 | 3.58 |
| Inpatient times | −0.0002 | 0.12 | −0.0000 | 0.04 | −0.0000 | 0.00 |

Abbreviations: CI, Contribution Index

S3 Decomposition of the concentration index of inequality in CHE for people with junior high school and above, 2013–2018

| **Variables** | **2013** | | **2015** | | **2018** | |
| --- | --- | --- | --- | --- | --- | --- |
|  | **Contribution to CI** | **Contribution to CI %** | **Contribution to CI** | **Contribution to CI %** | **Contribution to CI** | **Contribution to CI %** |
| Male | 0.0003 | −0.16 | −0.0008 | 0.55 | −0.0018 | 1.57 |
| Age |  |  |  |  |  |  |
| 51—60 years | −0.0135 | 7.56 | 0.0213 | −14.42 | 0.0028 | −2.41 |
| 61—70 years | −0.0079 | 4.44 | −0.0243 | 16.47 | −0.0114 | 9.94 |
| ≥ 71 years | −0.0118 | 6.64 | −0.0136 | 9.22 | −0.0124 | 10.81 |
| Junior high school−level education and above |  |  |  |  |  |  |
| Marital status | 0.0002 | −0.14 | 0.0018 | −1.23 | 0.0006 | −0.52 |
| Insurance | −0.0000 | 0.01 | 0.0004 | −0.27 | −0.0002 | 0.14 |
| Household size | −0.0010 | 0.55 | −0.0006 | 0.43 | −0.0028 | 2.47 |
| Economic status |  |  |  |  |  |  |
| Lower | −0.0393 | 22.08 | −0.0276 | 18.73 | −0.0202 | 17.58 |
| Middle | 0.0033 | −1.84 | 0.0012 | −0.83 | 0.0103 | −8.98 |
| Higher | 0.0012 | −0.66 | −0.0110 | 7.45 | −0.0023 | 1.97 |
| Highest | −0.0882 | 49.58 | −0.0605 | 41.04 | −0.0472 | 41.25 |
| Smoke | 0.0016 | −0.89 | 0.0005 | −0.31 | 0.0010 | 0.88 |
| Drink | 0.0025 | −1.38 | 0.0025 | −1.66 | 0.0022 | −1.90 |
| Disability | −0.0062 | 3.50 | −0.0046 | 3.10 | −0.0039 | 3.42 |
| Chronic diseases | −0.0042 | 2.35 | −0.0009 | 0.59 | −0.0098 | 8.53 |
| Outpatient | −0.0061 | 3.43 | −0.0016 | 1.09 | −0.0008 | 0.70 |
| Outpatient times | 0.0000 | −0.00 | −0.0125 | 8.47 | −0.0033 | 2.84 |
| Inpatient | −0.0034 | 1.93 | −0.0047 | 3.18 | −0.0031 | 2.74 |
| Inpatient times | 0.0005 | −0.29 | −0.0014 | 0.94 | 0.0040 | −3.52 |

Abbreviations: CI, Contribution Index

S4 Decomposition of the concentration index of inequality in CHE for people with elementary school and below, 2013–2018

| **Variables** | **2013** | | **2015** | | **2018** | |
| --- | --- | --- | --- | --- | --- | --- |
|  | **Contribution to CI** | **Contribution to CI %** | **Contribution to CI** | **Contribution to CI %** | **Contribution to CI** | **Contribution to CI %** |
| Male | −0.0003 | 0.19 | −0.0007 | 0.87 | −0.0003 | 0.63 |
| Age |  |  |  |  |  |  |
| 51—60 years | 0.0241 | −17.74 | 0.0225 | −29.78 | −0.0117 | −19.90 |
| 61—70 years | 0.0107 | −7.86 | 0.0107 | −14.21 | 0.0061 | −10.33 |
| ≥ 71 years | −0.0622 | 45.77 | −0.0628 | 83.18 | −0.04 | 70.57 |
| Marital status | 0.0064 | −4.71 | 0.0058 | −7.74 | 0.0050 | −8.53 |
| Insurance | 0.0005 | −0.33 | −0.0007 | 0.95 | 0.0006 | −0.96 |
| Household size | −0.0034 | 2.49 | −0.0050 | 6.67 | 0.0034 | −5.75 |
| Economic status |  |  |  |  |  |  |
| Lower | 0.0061 | −4.48 | 0.0099 | −13.11 | −0.0008 | 1.38 |
| Middle | −0.0015 | 1.08 | 0.0009 | −1.24 | −0.0006 | 1.10 |
| Higher | −0.0330 | 24.29 | −0.0204 | 27.09 | −0.0195 | 33.07 |
| Highest | −0.0794 | 58.43 | −0.0391 | 51.76 | −0.0225 | 38.32 |
| Smoke | −0.0009 | 0.64 | 0.0007 | −0.96 | 0.0001 | −0.21 |
| Drink | 0.0001 | −0.08 | 0.0000 | −0.05 | 0.0001 | 0.13 |
| Disability | 0.0007 | −0.54 | 0.0003 | −0.43 | −0.0003 | 0.52 |
| Chronic diseases | −0.0012 | 0.89 | −0.0016 | 2.18 | −0.0021 | 3.57 |
| Outpatient | 0.0014 | −1.06 | 0.0012 | −1.59 | 0.0007 | −1.23 |
| Outpatient times | −0.0010 | 0.73 | −0.0016 | 2.13 | 0.0006 | −1.09 |
| Inpatient | −0.0001 | 0.04 | −0.0001 | 0.07 | −0.0000 | 0.05 |
| Inpatient times | 0.0007 | −0.52 | 0.0013 | −1.77 | 0.0016 | −2.74 |

Abbreviations: CI, Contribution Index
